# A Direct Demonstration of Functional Differences between Subdivisions of Human V5/MT+

**DOI:** 10.1093/cercor/bhw362

**Published:** 2016-11-19

**Authors:** Samantha L. Strong, Edward H. Silson, André D. Gouws, Antony B. Morland, Declan J. McKeefry

**Affiliations:** 1 School of Optometry and Vision Science, University of Bradford, Bradford, West Yorkshire, BD7 1DP, UK; 2 Department of Psychology, York Neuroimaging Centre, University of York, York, YO10 5DD, UK; 3 Laboratory of Brain and Cognition, National Institute of Mental Health, Bethesda, USA; 4 Centre for Neuroscience, Hull-York Medical School, University of York, York, YO10 5DD, UK

**Keywords:** fMRI, psychophysics, transcranial magnetic stimulation, V5/MT+

## Abstract

Two subdivisions of human V5/MT+: one located posteriorly (MT/TO-1) and the other more anteriorly (MST/TO-2) were identified in human participants using functional magnetic resonance imaging on the basis of their representations of the ipsilateral versus contralateral visual field. These subdivisions were then targeted for disruption by the application of repetitive transcranial magnetic stimulation (rTMS). The rTMS was delivered to cortical areas while participants performed direction discrimination tasks involving 3 different types of moving stimuli defined by the translational, radial, or rotational motion of dot patterns. For translational motion, performance was significantly reduced relative to baseline when rTMS was applied to both MT/TO-1 and MST/TO-2. For radial motion, there was a differential effect between MT/TO-1 and MST/TO-2, with only disruption of the latter area affecting performance. The rTMS failed to reveal a complete dissociation between MT/TO-1 and MST/TO-2 in terms of their contribution to the perception of rotational motion. On the basis of these results, MT/TO-1 and MST/TO-2 appear to be functionally distinct subdivisions of hV5/MT+. While both areas appear to be implicated in the processing of translational motion, only the anterior region (MST/TO-2) makes a causal contribution to the perception of radial motion.

## Introduction

Moving visual stimuli elicit neural activity across an extensive network of areas in the human cerebral cortex (Watson et al. 1993; Dupont et al. 1994; Tootell et al. 1995; McKeefry et al. 1997; Smith et al. 1998; Culham et al. 2001). Within this network, human V5/MT+ (hV5/MT+) has come to be regarded as the cortical area most closely associated with the perception of visual motion (Zeki et al. 1991; Watson et al. 1993; Dumoulin et al. 2000). However, as its name implies, hV5/MT+ is not a single area but instead forms a complex, containing multiple areas that have been differentiated on the basis of differences in visual field representation and the receptive field (RF) sizes of constituent neurons (Dukelow et al. 2001; Huk et al. 2002; Amano et al. 2009). In this respect, hV5/MT+ mirrors its homolog in the monkey brain, which also comprises multiple visual areas. Importantly, these subdivisions in monkey V5/MT+ contain neurons that respond selectively to different types of moving stimuli (Desimone and Ungerleider 1986; Komatsu and Wurtz 1988; Tanaka et al. 1993; Nelissen et al. 2006; Kolster et al. 2009; Albright 1984, 2012). One subdivision, MT, contains directionally selective neurons that respond to many different types of motion (Lagae et al. 1994). Another subdivision, MST (Ungerleider and Desimone, 1986), has been further differentiated into a dorsal region (MSTd) where neurons respond preferentially to radial flow field stimuli and a ventrolateral region (MSTv or MSTl), which contains neurons that are more responsive to planar motion and are important in the generation of pursuit eye movements and object tracking (Saito et al. 1986; Mikami et al. 1986; Komatsu and Wurtz 1988; Duffy and Wurtz 1991a, 1991b; Tanaka et al. 1993, Eifuku and Wurtz 1998; Duffy, 1998).

Neuroimaging studies have managed to parcellate hV5/MT+ into at least 2 subdivisions (Dukelow et al. 2001; Huk et al. 2002; Amano et al. 2009), and the existence of additional areas seems highly likely (Kolster et al. 2010). The 2 subdivisions most consistently identified from posterior and anterior regions within hV5/MT+ and have been differentiated on various grounds including their respective representations of the ipsilateral and contralateral visual field, retinotopy, and population RF properties (Dukelow et al. 2001; Huk et al. 2002; Amano et al. 2009). Despite the different criteria employed by these studies, they all propose that the posterior subdivision of the hV5/MT+ complex is homologous with macaque area MT, while the more anterior region corresponds to MST. Differences do arise, however, in the nomenclature used across these studies to name the constituent components of hV5/MT+. Certain studies have adhered to the use of terms that reflect the functional differences first described in the monkey brain and refer to human MT and MST (Dukelow et al. 2001; Huk et al. 2002; Kolster et al. 2010). Others have employed the terms TO-1 and TO-2 to refer, respectively, to the posterior and anterior subdivisions that have been differentiated on the basis of retinotopic and population RF properties (Amano et al. 2009). In this study, we have adopted the hybrid terms MT/TO-1 for the posterior and MST/TO-2 for the anterior subdivisions to reflect their potential differentiation on the basis of both functional and retinotopic grounds. However, currently, the extent to which these subdivisions of hV5/MT+ are coextensive or to what degree they correspond to the functional properties displayed by monkey MT and MST (dorsal or ventral) is entirely not clear. Functional magnetic resonance imaging (fMRI) studies have demonstrated the existence of functional differences between constituent areas of the hV5/MT+ complex (Morrone et al. 2000; Kourtzi et al. 2002; Smith et al. 2006; Kolster et al. 2010). One observed difference, consistent with the functional specializations reported for monkey MT and MST, is that the anterior subdivision of hV5/MT+, MST/TO-2, is more responsive to radial motion or optic flow stimuli and appears to be more specialized for encoding the global flow properties of complex motion stimuli, compared with its posterior counterpart MT/TO-1 (Smith et al. 2006). Along similar lines, Wall et al. (2008) reported that human MST/TO-2, unlike MT/TO-1, exhibits adaptation to optic flow stimuli further emphasizing the sensitivity of the former to more complex optic flow stimuli.

The purpose of this study was to investigate the existence of functional differences between the subdivisions of hV5/MT+ and demonstrate the extent to which the perception of different kinds of motion stimuli is critically dependent on neural activity within these subdivisions. While fMRI experiments can identify cortical areas that exhibit the appropriate response properties that correlate with a given perceptual function, it is necessary to use interventional techniques to demonstrate causality. One such technique is transcranial magnetic stimulation (TMS). TMS can be used to induce transient and localized disruption of human cortical function (Pascual-Leone et al. 2000; Walsh and Cowey 2000). Motion perception has proven to be very amenable to study using TMS (Beckers and Homberg 1992; Hotson et al. 1994; Beckers and Zeki 1995; Anand et al. 1998; Walsh et al. 1998; Cowey et al. 2006; Laycock et al. 2007; McKeefry et al. 2008), and in this study, we deployed TMS to selectively disrupt neural activity in areas MT/TO-1 and MST/TO-2 having first identified these regions of interest (ROIs) using previously employed fMRI localizers. We then measured the effects of targeted disruption to MT/TO-1 and MST/TO-2, while human observers performed directional judgment tasks for different kinds of motion stimuli (translational, radial, and rotational). Our aim was to provide evidence of a direct causal relationship between the neural activity within areas MT/TO-1 and MST/TO-2 and the perception of different kinds of motion stimuli.

## Materials and Methods

### Participants

Nine participants (age range 21–46 years; mean age 29.3 years; 6 male) took part in this study, all of whom had normal or corrected-to-normal vision at the time of testing and had no history of neurological or psychiatric disorders. Experiments were conducted in accordance with the Declaration of Helsinki and were approved by both York Neuroimaging Centre Ethics Committee and the University of Bradford Ethics Committee.

### MRI and Analysis

Functional T2* MR images were acquired using a GE 3-Tesla Sigma Excite HDX MRI scanner. Gradient-recalled echo pulse sequences were used to measure blood oxygenation level-dependent (BOLD) signal as a function of time (TR = 3000 ms, TE = 29 ms, FOV = 192 cm, 128 × 128 matrix, 39 contiguous slices, 1.5 × 1.5 × 1.5 mm^3^, interleaved slice order with no gap). A 16-channel phased-array half-head coil positioned at the occipital pole of the subject was used to measure MR signal focused on the visual cortex. A high-resolution T1-weighted 3D anatomical data set was used for co-registration of functional and structural data. This was acquired using an 8-channel phased-array full-head coil (TR = 7.8 ms, TE = 3 ms, TI = 450 ms, FOV = 290 × 290 x 276, 256 × 256 × 176 matrix, flip angle = 20°, 1.13 × 1.13 × 1.0 mm^3^).

The data obtained from these functional scans were analyzed using BrainVoyager QX software (Version 3.0, Brain Innovation). Preprocessing of this data included spatial smoothing (3 mm Gaussian kernel, full width at half maximum), 3D motion correction, slice scan timing correction, and high-pass (GLM-Fourier) temporal filtering (0.01 Hz). Multiple linear regression was then applied to the data allowing contrasts to be made between moving–static conditions within each subject across multiple runs. Hemodynamic responses were corrected appropriately for neurovascular lag (Boynton et al. 1996).

### Identification and Localization of Target and Control Sites

**Figure 1.**
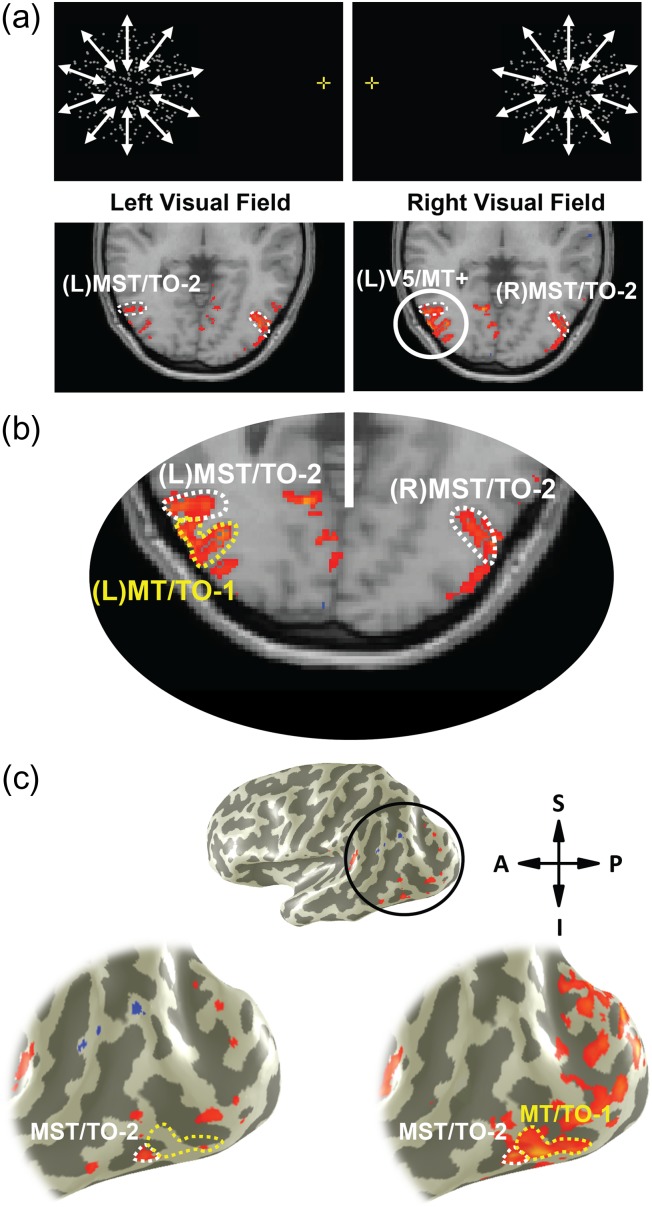
Stimulus specification and identification of MT/TO-1 and MST/TO-2. (*a*) (Top row) Example stimuli showing dots presented in either the left or right visual field. (Bottom row) Axial fMRI data from 1 representative subject (S3) showing BOLD signal (*P* < 0.001) generated by moving versus static functional localizers presented in both the left and right visual field (averaged across 4 runs). Anterior MST/TO-2 (white dotted line) can be seen relative to whole V5/MT+ complex in both hemispheres and is identified as the anterior portion of the complex activated by ipsilateral stimuli. (*b*) Magnified view of posterior occipital lobe in the same subject when viewing dots in the right visual field demonstrating the identification of MT/TO-1. Here, left MT/TO-1 (yellow dotted line) is shown as the subtraction of the MST/TO-2 ipsilateral activation (white dotted line) from the whole V5/MT+ complex activated by contralateral stimulation. (*c*) Areas MST/TO-2 and MT/TO-1 shown as increases in BOLD signal superimposed onto 3D inflated surfaces of the left cerebral hemisphere of subject S3. The black circle highlights the magnified area of the 2 images, with the left image showing ipsilateral activation of MST/TO-2 (white dotted line) produced when dots were viewed in the LVF. Similarly, the right image shows identification of MT/TO-1 (yellow dotted line) when MST/TO-2 is subtracted from the full contralateral activation of V5/MT+ produced by viewing dots in the right visual field.

Two subdivisions of hV5/MT+, designated MT/TO-1 and MST/TO-2, were identified using techniques similar to those described previously (Dukelow et al. 2001; Huk et al. 2002). In a block-design paradigm, subjects viewed 15-s periods of moving and static dots, interspersed with blank intervals. The moving dots were restricted to a 15° (diameter) circular aperture, the center of which was horizontally displaced by 15° from the central fixation point in either the left or right visual field depending on the trial. The aperture contained 300 white dots (each ~0.2° in diameter) presented on a black background and moved at a speed of 7°/s radially inwards or outwards with the direction alternating every second. The static dots were restricted to the same circular aperture as the moving dots. Each trial contained 5 repeats of the cycle and throughout the trial subjects fixated on a central fixation point (Fig. 1*a*). The more anteriorly located MST/TO-2 was localized in each hemisphere by identifying activations in the hemisphere ipsilateral to stimulation of either the right or left visual field (LVF) (Fig. 1*b*). MT/TO-1 was then identified by subtracting the anterior MST/TO-2 activity from the whole hV5/MT+ complex activation found for contralateral presentations. By contrast moving with static activity from the MST/TO-2 localizer, ipsilateral increases in BOLD signal were found in 17/18 hemispheres. Of these 17 hemispheres, stimuli presented in the LVF produced a significant cluster of activation in anterior left hV5/MT+ (left MST/TO-2), and stimuli in the right visual field produced activation in right anterior hV5/MT+ (right MST/TO-2). This is consistent with the findings of previous literature that has characterized the large RF sizes of these anterior regions (Amano et al. 2009).

**Figure 2.**
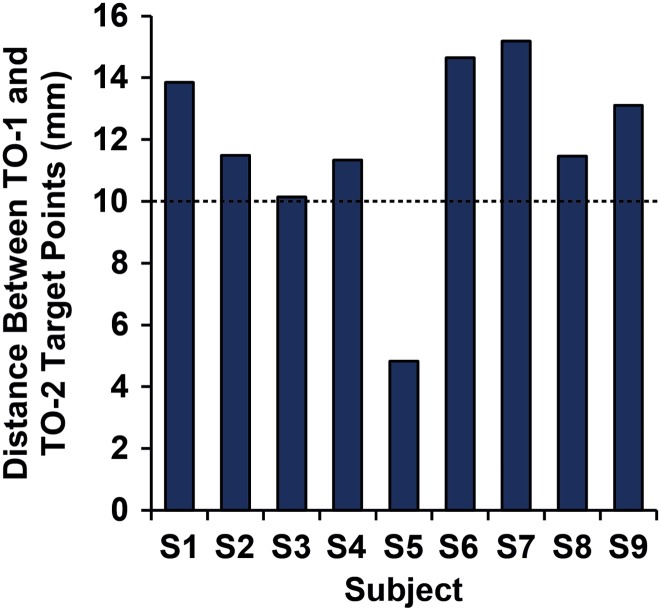
Bar chart showing Euclidean distance (in millimeters) between MT/TO-1 and MST/TO-2 in the right hemisphere for each subject. The black dashed line denotes the 10 mm separation criterion.

Although some local spread of the TMS magnetic field occurs across tissue adjacent to the targeted site, previous research has shown that the differential effects of TMS are measurable in target sites with centroids as little as 10 mm apart in human cortex (cf. Pitcher et al. 2009; Silson et al. 2013). Following this, 10 mm was used as the minimum criteria for distance between target points in each subject. Target points for each of our sites of interest were created by overlaying the functional data onto a 3D structural scan and creating target points for both MT/TO-1 and MST/TO-2 based on their respective centers of mass. The Euclidean distance (d) between these target points was then computed (Fig. 2). In the right hemisphere, it was found that MT/TO-1 and MST/TO-2 were at least 10 mm apart in 8/9 hemispheres. One subject (S5) did not meet the minimum criteria for inter-target distance and so was removed from the subset of subjects that were carried forward to take part in the TMS experiment.

**Figure 3.**
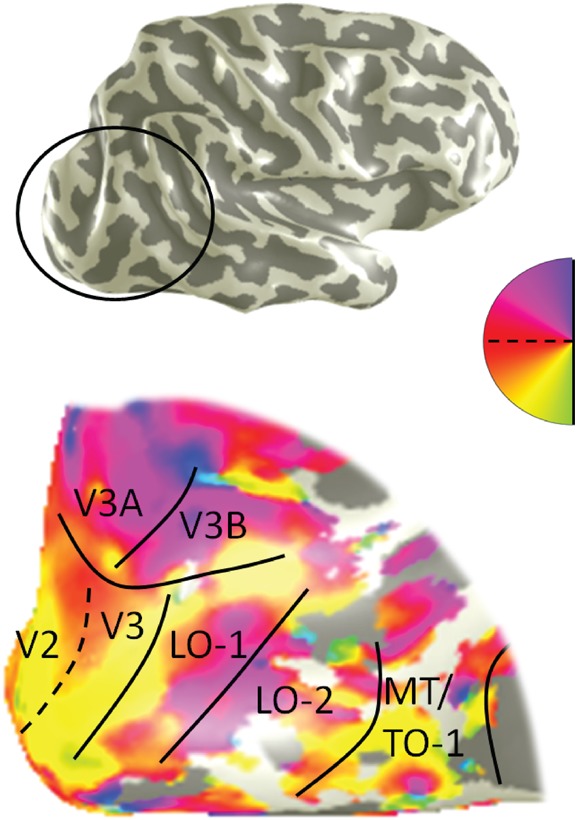
Location of the LO-1 control stimulation site. In subject S3, a portion of the lateral surface of the posterior occipital lobe is highlighted. This region is magnified in the lower part of the diagram and shown as an inflated 3D mesh with a pseudocolor representation of the visual field.

Retinotopic mapping techniques (Sereno et al. 1995; DeYoe et al. 1996; Engel et al. 1997; Wandell et al. 2007) using a 90° anticlockwise rotating wedge (contrast reversal rate 6 Hz) and an expanding annulus (≤15°radius), both lasting 36 s per cycle, were used to identify the control site (LO-1) in each subject. Consistent with previous data (Larsson and Heeger 2006; Silson et al. 2013), LO-1 was found adjacent to V3d representing the contralateral lower visual field posteriorly and the contralateral upper visual field anteriorly (Fig. 3). Cortical area LO-1 was chosen as a control site because it lies in close proximity to areas MT/TO-1 and MST/TO-2, but in contrast to these areas LO-1 has no known role in the processing of visual motion, appearing instead to be involved in processing orientation information related to the recognition of objects (Larsson and Heeger 2006; Silson et al. 2013). The use of this control site should determine whether there are any effects of proximity to TMS on performance. It should also allow us to confirm that any effects found from applying TMS to the target ROIs are not simply due to the general effect of applying TMS to the extra-striate visual cortex.

We did attempt to differentiate MT/TO-1 and MST/TO-2 using retinotopic criteria, but we found that while MT/TO-1 did seem to possess retinotopy, the maps obtained from MT/TO-2 were less reliable. This is consistent with the results of Huk et al. (2002) and presumably is a reflection of the increased RF sizes for MST/TO-2 that are less likely to be shown to be retinotopic by the spatial and temporal parameters of the standard rotating wedge and ring stimuli employed here (see Kolster et al. 2010 for a discussion on this point).

### Co-registration of fMRI and TMS Target Sites

The target points for repetitive TMS (rTMS) delivery were chosen as the center of mass coordinates within the areas identified as MT/TO-1, MST/TO-2, and LO-1 following the fMRI experiments described above. All of the target ROIs selected for TMS were located in the right cerebral hemisphere, and the mean Talairach coordinates for each of these sites are given in Table 1. Note that the target sites for areas in this study were identified primarily on the basis of the ipsilateral versus contralateral responses to motion stimuli. The control site, LO-1, was identified on the basis of its previously described retinotopy (Larsson and Heeger 2006; Silson et al. 2013). Table 1 also includes Talairach coordinates for MT/TO-1 and MST/TO-2, which in previous studies have identified on both retinotopic and functional grounds (Dukelow et al. 2001; Kolster et al. 2010). Despite the different criteria employed, there appears to be a close degree of correspondence across the studies in terms of the location of the centers of these ROIs.

**Table 1:** Table comparing average Talairach coordinates for MT/TO-1 and MST/TO-2 from this study with coordinates from Dukelow et al. (2001) and Kolster et al. (2010)

	MT/TO-1			MST/TO-2		
	x	*y*	*z*	*x*	*y*	*z*
Right Hemisphere						
This study	42 ± 2.7	−76 ± 3.0	−3 ± 7.4	43 ± 4.1	−69 ± 6.7	0 ± 8.8
Dukelow et al. (2001)	44 ± 3	−64 ± 7	5 ± 4	45 ± 3	−60 ± 5	5 ± 4
Kolster et al. (2010)	46	−78	6	44	−70	5
Left Hemisphere						
This study	−46 ± 2.3	−79 ± 2.7	−1 ± 8.9	−47 ± 5.7	−71 ± 5.3	−2 ± 9.1
Kolster et al. (2010)	−48	−75	8	−45	−67	6

Following identification of the 3 target points in 3D space, co-registration between each subject's head and their structural scans was achieved using a 3D ultrasound digitizer CMS30P (Zebris) in conjunction with BrainVoyager QX (McKeefry et al. 2008). This method creates a local spatial coordinate system that is able to link the spatial positions of ultrasound transmitters on the subject and the coil with prespecified fiducials on the 3D representations.

### Stimuli and Psychophysical Procedures

**Figure 4.**
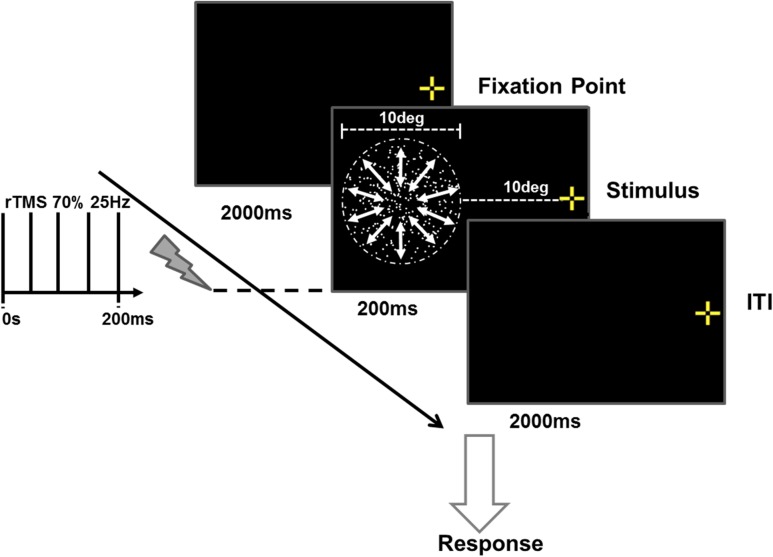
Psychophysical procedure for direction discrimination task using radial motion as the example stimulus. In the rTMS trials, pulse trains were delivered synchronously with the onset of stimuli and persisted for an equivalent duration (200 ms). Following stimulus offset, the subjects reported the perceived motion direction relevant to the task (up/down for translational motion, in/out for radial motion, clockwise/anticlockwise for rotational motion) by an appropriate key press.

All motion stimuli were displayed on a high-resolution cathode ray tube monitor with a refresh rate of 75 Hz (Mitsubishi DiamondPro 2070SB). Stimuli were generated using Psychophysics Toolbox Version 3 (Brainard 1997; Pelli 1997; Kleiner et al. 2007) in 32-Bit MATLAB (Version 7.6.0; The MathWorks Inc., Natick, MA, 2008). Dot stimuli were restricted to a 10° circular aperture containing 300 white dots on a black background. Each dot subtended 0.2° of visual angle (dot density ~3.82/deg^2^), and all dots moved at a speed of 7°/s regardless of direction. The center of this aperture was horizontally displaced by 15° to the left of the fixation point (Fig. 4). Three kinds of motion stimulus were used; 1) translational motion—where the dots moved either up or down, 2) radial motion—where the dots moved outwards or inwards from the center of the aperture, and 3) rotational motion—where the dots rotated in a clockwise or counterclockwise about the center of the aperture.

In each type of motion stimulus, a low, but detectable, percentage of dots moved in a coherent direction (signal dots), while the rest moved randomly (noise dots). Participants were required to identify the coherent direction of the signal dots using a 2-alternative forced choice (2AFC) paradigm for 3 different conditions: translational coherent dots (up or down), radial coherent dots (inward or outward), and rotational coherent dots (clockwise or anticlockwise). Subjects were instructed to record their decision regarding the direction of the signal dots using an appropriate button on the keyboard as quickly and as accurately as possible. Our decision to employ the 3 different motion stimuli tasks was motivated by earlier single-unit monkey neurophysiology (Duffy and Wurtz 1991a, 1991b) and human neuroimaging studies (Smith et al. 2006). These studies have shown that different selectivities (particularly for radial motion stimuli) exist across the subdivisions of human and monkey V5/MT+, which we wished to exploit using similar stimulus types. For each participant, and separately for each motion stimulus type, preliminary psychophysical measurements were used to establish the proportion of dot coherence required to result in correct motion discrimination 75% of the time. The psychophysical data were then fitted by a 2-parameter logistic function of the form:

(1) $$P\left(x\right)=[{1+\exp\left\{\delta \left(\theta -x\right)\}\right]}^{-1}$$

where *x* represents the stimulus value (coherence), *P(x)* is the response probability at *x*, *δ* is the slope parameter, and *θ* represents the threshold parameter corresponding to the stimulus level at which response probability is 75%. These subject- and stimulus-specific coherence values (Table 2) were used in the main TMS experiments where subjects performed ~100 trials for each condition in each task. Trials were removed if the subject took longer than 3 s to respond. Overall <3% of trials were removed from the complete data set.

**Table 2:** Table showing individual 75% thresholds for all 3 types of moving dot pattern for each subject

Subject	Translational (%)	Radial (%)	Rotational (%)
S1	22.6	13.6	10.9
S2	27.4	24.4	13
S3	16.0	10.1	7.9
S4	20.9	13.0	6.6
S6	26.3	15.4	10.2
S7	39.8	20.2	4.4
S8	29.4	24.4	8.7
S9	35.1	23.7	5.0
Average	27.2 ± 7.7	18.1 ± 5.8	8.3 ± 3.0

Note: Table also demonstrates average values ± standard deviation.

### TMS Protocol

During the task, participants viewed a centrally placed fixation cross with their right eye (left eye occluded) from a distance of 57 cm. The center of a 10° (diameter) stimulus was placed 15° horizontally relative to the fixation cross in the LVF. This was done to minimize any involvement of ipsilateral V5/MT+ in the performance of the motion discrimination tasks. In these experiments, TMS was delivered to the target sites in the right hemisphere, leaving their counterparts in the left hemisphere functioning normally. Amano et al. (2009) have demonstrated that the RFs of hV5/MT+ neurones can extend well beyond the vertical meridian into the ipsilateral (in this case the left) visual field. Our stimulus placement was therefore an attempt to minimize contributions from the ipsilateral non-stimulated motion area. Similar reasoning lies behind the choice of stimulus size (10° diameter) for the TMS experiments, in that larger stimulus sizes extending towards the midline would also allow the involvement of neurons with large RFs from the undisrupted ipsilateral hV5/MT+. The choice of stimulus size is at the first glance at odds with results from monkey single-unit neurophysiology, where studies have shown that MSTd neurons give weak responses to relatively small stimuli (Komatsu and Wurtz 1988). However, more recent neuroimaging studies in human have clearly shown that activation of human MST/TO-2 (and its differentiation from MT/TO-1) can be achieved for stimuli of sizes 8°x 8° and greater (Becker et al. 2008).

In the combined TMS and psychophysical experiments, the onset of the motion stimulus was synchronous with onset of a train of 5 biphasic (equal relative amplitude) rTMS pulses (Fig. 4). Previous results had demonstrated that this temporal configuration was most effective at inducing effects in hV5/MT+ (McKeefry et al. 2008). These pulses were applied to the participant's scalp using a figure-of-eight coil (50 mm diameter) connected to a Magstim Super Rapid 2 stimulator (Magstim, Wales, UK). The rTMS trains were applied at a frequency of 25 Hz, at a level of 70% of the maximum output. Participants undertook 2 blocks of the motion task for each TMS site and condition. Only 1 condition was tested in each session, and the order of presentation of conditions was counterbalanced across participants.

### Statistical Analysis

Statistical analysis of the results was performed using the SPSS software package (IBM). Repeated-measures analyses of variance (ANOVAs) were calculated across all conditions (baseline, MT/TO-1, MST/TO-2, and LO-1 control) for each of the 3 tasks individually (translational, radial, and rotational). When a significant main effect was present, pairwise comparisons were applied to the data sets (Bonferroni corrected for multiple comparisons). The assumption of normal distribution was confirmed with Mauchly's Test of Sphericity. If this assumption was met (i.e., sphericity is nonsignificant), then the ANOVA was calculated assuming sphericity; however, if the assumption was violated, the degrees of freedom (dF) would be corrected to allow appropriate interpretation of the *F* value of the ANOVA. These dF corrections included the Greenhouse–Geisser correction when sphericity was less than 0.75, and Huynh–Feldt correction when sphericity exceeded 0.75.

## Results

**Figure 5.**
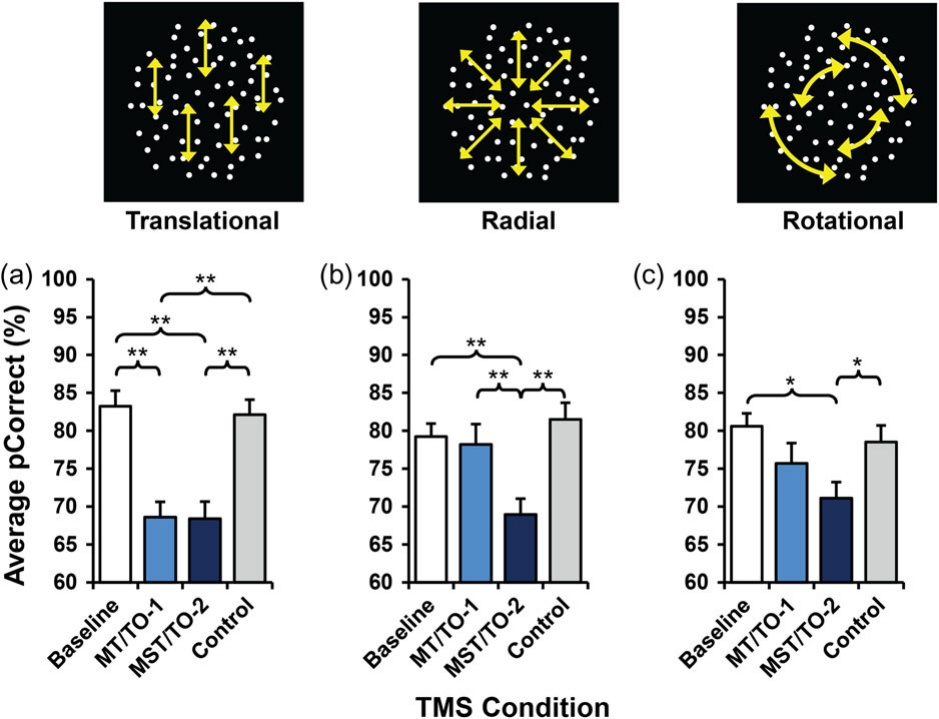
Bar charts showing average proportion correct for translational (*a*), radial (*b*), and rotational (*c*) motion tasks (a low value suggests the task was found to be more difficult). Single asterisks (*) highlight significant differences at 0.05 level; double asterisks (**) highlight significant differences at 0.01 level. Error bars show SEM.

Percent correct (pCorrect) is the main dependent variable measured within this experiment. This variable quantifies the subject- and condition-specific variance in performance around a 75% threshold as a function of the task performed and TMS stimulation condition. Applying rTMS to MT/TO-1 and MST/TO-2 during motion coherence direction discrimination tasks appears to produce effects that are task-specific (Fig. 5). Significant main effects of experimental condition on performance were found for all 3 motion direction discrimination tasks: translational (*F*(3,21) = 30.35, *P* < 0.001), radial (*F*(3,21) = 13.40, *P* < 0.001), and rotational (*F*(3,21) = 8.34, *P* = 0.001).

For translational motion, pairwise comparisons showed that application of TMS to both MT/TO-1 and MST/TO-2 produced significant reductions in the ability of subjects to determine the direction of motion of the dots relative to both baseline and control conditions (MT/TO-1 versus baseline, *P* = 0.003; MT/TO-1 versus control, *P* = 0.007; MST/TO-2 versus baseline, *P* = 0.001; MST/TO-2 versus control (LO-1), *P* = 0.002). No other pairwise comparisons were found to be significant (*P* = 1.00 in all cases). Clearly, for the discrimination of the translational motion direction, the consequences of disruption by TMS are the same for the 2 areas, indicating that neural activity in both MT/TO-1 and MST/TO-2 is essential for the perception of such stimuli.

In contrast, for radial motion, there was a significant differential effect on perception when TMS was applied to MT/TO-1 and MST/TO-2. When MT/TO-1 was targeted, there was no significant effect on performance (*P* = 1.00), whereas application of TMS to MST/TO-2 resulted in a decrease in subjects’ ability to perceive radial motion relative to baseline and the control site (MST/TO-2 versus baseline, *P* = 0.005; MST/TO-2 versus control, *P* = 0.005). Importantly, there is a significant dissociation between the effects of TMS on the perception of radial motion between MT/TO-1 and MST/TO-2 (MST/TO-2 versus MT/TO-1, *P* = 0.007) demonstrating that neural activity within area MST/TO-2, but not MT/TO-1, is required for performance of the radial motion discrimination task.

For the rotational motion task, application of TMS to MST/TO-2 significantly reduced performance relative to baseline and control (MST/TO-2 versus baseline, *P* = 0.017; MST/TO-2 versus control, *P* = 0.035). Application of TMS to MT/TO-1 produced no significant effects (MT/TO-1 versus baseline, *P* = 0.289). However, while there are clear deficits relative to the baseline and control conditions when TMS is applied to MST/TO-2, comparisons with performance when TMS is applied to MT/TO-1 fall short of showing complete dissociation between the 2 areas (MT/TO-1 versus MST/TO-2, *P* = 0.687). No other comparisons were found to be significant (baseline versus control, *P* = 0.371; MT/TO-1 versus control, *P* = 1.00).

**Figure 6.**
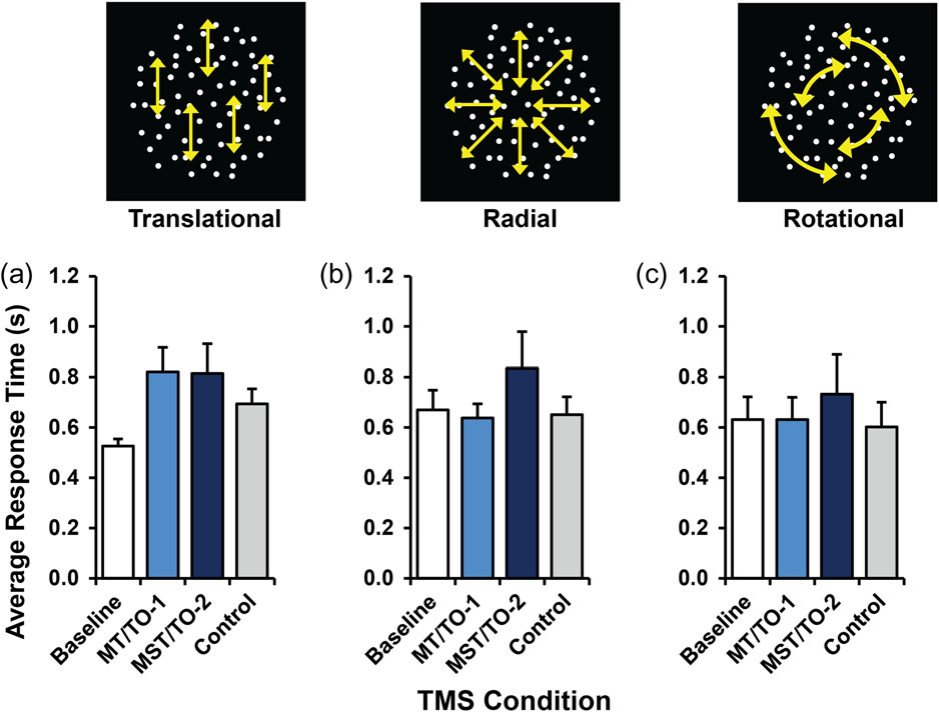
Bar chart showing average response time for translational (*a*), radial (*b*), and rotational (*c*) motion tasks. The data show no significant differences at 0.05 level. Error bars show SEM.

Response times were recorded for every trial in all 3 tasks. A significant main effect of response time was found for translational motion (*F*(3,21) = 4.24, *P* = 0.017); however, pairwise comparisons failed to identify any significant differences between conditions (baseline versus MT/TO-1, *P* = 0.189; baseline versus MST/TO-2, *P* = 0.336; baseline versus control, *P* = 0.307; MT/TO-1 versus control, *P* = 0.607; all other comparisons, *P* = 1.00). No significant main effects of response time were found for either radial (*F*(3,21) = 1.88, *P* = 0.165) or rotational motion (*F*(3,21) = 0.99, *P* = 0.416), showing that response time did not differ significantly across any of the TMS conditions in either of those tasks (Fig. 6). However, it is noteworthy that longer reaction times occurred under conditions where discrimination deficits were found—the opposite of a speed–accuracy trade-off.

Across all tasks and all motion conditions, there were no significant differences between baseline performance and performance during TMS of the control site. This similarity supports the decision to use this site (LO-1) as a suitable visual cortex control, and we can therefore conclude that any experimental effects found are not a result of confounding variables associated with general application of TMS as this would also affect the performance associated with control site. No significant effects were found for response times across radial and rotational motion, confirming that the response times will not have had any confounding effects on the performance of the subject.

## Discussion

This study has demonstrated that when TMS is used to disrupt neural activity within 2 major subdivisions of hV5/MT+, MT/TO-1, and MST/TO-2, dissociable effects on the perception of different kinds of moving stimuli can be induced in human observers. The application of TMS to either MT/TO-1 or MST/TO-2 impairs significantly the perception of translational motion, but only disruption of MST/TO-2 induces significant deficits in the perception of radial flow patterns. Previous neuroimaging experiments have successfully managed to distinguish between these subdivisions of hV5/MT+ on the basis of retinotopy and RF properties (Dukelow et al. 2001; Huk et al. 2002; Amano et al. 2009). In addition, differences in the response selectivities of MT/TO-1 and MST/TO-2 to different kinds of motion stimuli have also been demonstrated (Morrone et al. 2000; Smith et al. 2006; Wall et al. 2008; Kolster et al. 2010; Pitzalis et al. 2013). This study provides further evidence of functional differences between the constituent subdivisions of hV5/MT+ by establishing the existence of causal dependencies between neural activity within these subdivisions and the perception of different kinds of moving stimuli.

Our results demonstrate that the anterior subdivision of hV5/MT+, area MST/TO-2, appears integral to the perception of radial flow patterns. In this respect, human MST/TO-2 appears functionally similar to monkey MSTd, the neurons of which exhibit a similar response selectively (Saito et al. 1986; Tanaka and Saito 1989; Tanaka et al. 1989; Duffy and Wurtz 1991a, 1991b; Lagae et al. 1994). However, this is directly at odds with the previous proposals of homology between human MST/TO-2 and monkey MSTv made on the basis of similarities between their RF characteristics (Amano et al. 2009). The justification for our proposal that MST/TO-2 is more closely allied to MSTd rather than MSTv is based on the functional similarities between MST/TO-2 and MSTd, coupled with its functional differences with MSTv, where neurons are unresponsive to radial flow stimuli and more important for the generation of smooth pursuit eye movements (Eifuku and Wurtz 1998). These conflicting findings relating to the RF and functional properties highlight the difficulties in establishing clear homologies between the subdivisions of human and monkey V5/MT+. This is further complicated by the fact that other studies of hV5/MT+ suggest there may be at least 4 separate representations of the visual field [MT, pMSTv, pFST, and pV4t {p = putative}] within hV5/MT+ (Kolster et al. 2010). The Talairach coordinates for MT and pMSTv as defined by Kolster et al. (2010) agree very closely with our locations of MT/TO-1 and MST/TO-2, respectively (Table 1). The use of the term pMSTv by Kolster et al. (2010) clearly suggests homology with monkey MSTv and, in view of the coinciding cortical locations, also with our MST/TO-2. However, Kolster et al (2010) define pMSTv purely in terms of retinotopic criteria. While their data highlight that pMSTv is responsive to motion and less so to shape, no data are presented to assess whether it adheres to the functional criteria (e.g. lack of selectivity to radial flow stimuli) that have previously been used to differentiate MSTv and MSTd in monkeys (Desimone and Ungerleider 1986; Komatsu and Wurtz 1988; Tanaka et al. 1993). Therefore, a link between MST/TO-2 and MSTd cannot be conclusively ruled out until we have a clearer understanding of the functional properties of human pMSTv.

It has been argued that the 4-component composition of hV5/MT+ revealed by Kolster et al. (2010) is more consistent with the structure of monkey V5/MT+ and, moreover, constitutes a key organizational feature of V5/MT+ across all primates (Nelissen et al. 2006; Kolster et al. 2010). Similar to the monkey brain, the multiple visual field maps present in human V5/MT+ have been linked to the analysis of different aspects of motion processing. They are hypothesized as forming the basis of separate processing pathways, emanating from V5/MT, which are involved in the analysis of different kinds of moving stimuli (Xiao et al. 1997; Kourtzi et al. 2002; Kolster et al. 2009). In this study, we were unable to find evidence of separate ventral retinotopic maps corresponding to FST and V4t. However, the absence of evidence does not constitute evidence of absence, and it may be that our retinotopic mapping protocols are simply not suited to revealing all of the individual maps that may be present in hV5/MT+. For example, the constituent subdivisions of hV5/MT+ are thought to share a foveal confluence (Kolster et al. 2010); it is likely that the size of wedge employed for this experiment may have been too large to accurately distinguish between phase reversals in foveal cortex (Schira et al. 2009). It may very well prove to be the case that the subdivision of V5/MT+ into 4 separate areas, rather than just 2, is the common organization feature across all primate brains.

Our results are consistent with the previous fMRI studies that have examined functional differences between the subdivisions of hV5/MT+ defined by the same criteria (Dukelow et al. 2001; Huk et al. 2002; Amano et al. 2009). The data show that human MST/TO-2 is differentially responsive to the basic components of optic flow (Smith et al. 2006; Wall et al. 2008; Pitzalis et al. 2013). However, the precise pattern of stimulus selectively reported for human MST/TO-2 is not consistent across all studies. Smith and colleagues (Smith et al. 2006; Wall et al. 2008) found human MST/TO-2 to be responsive to radial and rotational flow patterns, while Pitzalis et al. (2013) showed the same area to be most responsive to radial motion but less so to rotational stimuli. The reasons for these discrepancies are not clear, but the results described by Pitzalis et al. (2013) appear to be more in line with the pattern of deficits induced by TMS in this study where we have failed to reveal a complete dissociation between the contribution of MT/TO-1 and MST/TO-2 to the perception of rotational motion. This finding is consistent with neuropsychological case studies (Beardsley and Vaina 2005) which point to the existence of separate cortical loci for the neural activity that supports the perception of radial and rotational motion stimuli.

Radial motion stimuli are considered as having particular significance in that the expansion/contraction from/to a central point provides an important cue for the guidance of self-motion and for ecologically important visual tasks within visual environments (Gibson 1950; Warren and Hannon 1988; Warren and Rushton 2009). Evidence from the monkey brain suggests that neurons in area MSTd play a key role in the analysis of this information (Tanaka and Saito 1989; Duffy and Wurtz 1991a, 1995), and that electrical stimulation of this area can bias directional judgments of self-motion made by monkeys (Britten and Van Wezel 1998). Reports that regions within hV5/MT+ exhibit similar response selectivity for radial motion stimuli have led to the proposal that area MST/TO-2 plays a similar role in the encoding of self-motion in the human brain (Morrone et al. 2000; Pitzalis et al. 2013). In addition to MST/TO-2, radial flow patterns have also been shown to elicit responses in other brain areas such as V3A, V3B, V6, and the intraparietal sulcus (Morrone et al. 2000; Smith et al. 2006; Wall and Smith, 2008; Cardin et al. 2012). MST/TO-2 possibly forms just 1 component area within a hierarchical processing network comprising multiple cortical areas within which increasingly more complex analyses allow the extraction of behaviorally relevant information from radial flow stimuli (Wall and Smith, 2008).

TMS can alter the signal:noise ratio of neural activity within a cortical region in various ways that will result in functional impairments. Current TMS research has yet to agree on exactly how the effects of TMS are mediated within the cortex. Some studies posit that TMS induces neural noise (Ruzzoli et al. 2010), others posit that it reduces neural signal (Harris et al. 2008), while another school of thought proposes that TMS may be both reducing signal and increasing noise at the same time (Allen et al. 2007). Regardless of how TMS disrupts cortical function, we demonstrate that measurable and specific functional deficits are induced by the delivery of TMS to particular target sites. We are able to demonstrate that there is a causal dependence between different aspects of motion perception and neural activity within separate subdivisions of hV5/MT+. Crucially, the functional deficits induced by the application of TMS to areas MT/TO-1 and MST/TO-2 do not constitute a double dissociation. Such a finding would have carried the implication that the analysis of translational and radial motion occurs independently within MT/TO-1 and MST/TO-2. Instead, disruption to both MT/TO-1 and MST/TO-2 induces deficits in the perception of translational motion, while the perception of radial motion is only affected by disruption of MST/TO-2. Rather than independent processing, this suggests a more serial form of processing where information is passed on from MT/TO-1 and subsequently subjected to more complex analysis from which sensitivity to radial flow emerges only at the level of MST/TO-2. Neurons in monkey MT have smaller RFs than those found in MST which as a result are able to integrate motion signals over much larger spatial extents (Saito et al. 1986). Mechanisms supporting this kind of transformation have been described in monkey MST (Yu et al. 2010) where sensitivity to optic flow has been found to be based on local selectivity to translational motion within subregions of the large RFs of MST neurons. This localized response is then combined across the whole RF to generate global sensitivity to optic flow (Yu et al. 2010). Similar local versus global RF sensitivities may explain the functional deficits found in human MST/TO-2 in this study.

## Conclusion

This study has provided direct evidence of causal links between neural activity in different subdivisions of hV5/MT+ and the perception of different kinds of motion stimuli. The results provide further confirmation of what has long been suspected; namely hV5/MT+ comprises multiple visual areas that are separable, not only on retinotopic but also on functional bases. In this respect, motion processing in the human brain may be organized along similar lines to that found in the monkey and is based on a hierarchy of retinotopically distinct visual areas, the neurons of which possess increasing RF size, response selectivity, and processing complexity (Orban 2008). Areas MT/TO-1 and MST/TO-2 in the human brain, like MT and MSTd in the monkey, may provide the origins of different cortical processing networks that are involved in the analysis of planar motion and radial flow, respectively.
